# Modified multiple papilla full flap combined with tunneling and de-epithelialized gingival graft to manage severe gingival recession in mandibular incisors: literature review and case report

**DOI:** 10.1093/jscr/rjae479

**Published:** 2024-08-06

**Authors:** Quan Dong Ta, Nhan Van Vo, Hanh My Le Bui, Trung Dang Quoc Tran, Viet Hoang

**Affiliations:** Department of Implantology, Nhan Tam Dental Maxillofacial Speciality Hospital, 3/2 Street, District 10, Ho Chi Minh City, 70000, Vietnam; Department of Prosthodontics, Faculty of Odonto-Stomatology, Hong Bang International University, 215 Dien Bien Phu Street, Ward 15, Binh Thanh District, Ho Chi Minh City, 70000, Vietnam; Department of Implantology, Nhan Tam Dental Maxillofacial Speciality Hospital, 3/2 Street, District 10, Ho Chi Minh City, 70000, Vietnam; Department of Implantology, Nhan Tam Dental Maxillofacial Speciality Hospital, 3/2 Street, District 10, Ho Chi Minh City, 70000, Vietnam; Department of Orthodontics and Pedodontics, Faculty of Dentistry, Van Lang University, 69/68 Dang Thuy Tram, Ward 13, Binh Thanh District, Ho Chi Minh City, 70000, Vietnam

**Keywords:** connective tissue, recession, tunnelling, multiple papilla full thickness flap, gingival draft, orthodontic treatment

## Abstract

Gingival recession is a common condition in adults, causing esthetic issues or tooth sensitivity in patients. Gingival recession in the lower incisors area after orthodontic treatment is often difficult to predict, due to anatomical factors such as thin phenotype, shallow vestibular depth, and low frenum attachment. A young male patient presented to the clinic with severe Cairo Class I gingival recession at tooth 41 after 2 years of orthodontic treatment. Surgery combining tunneling technique and multiple papilla full thickness flap was performed to treat the patient after endodontic treatment. The clinical case was followed up to monitor gingival margin recovery one year post-surgery. The clinical case introduces a treatment method for severe localized gingival recession in the lower anterior region of a single tooth.

## Introduction

Gingival recession (GR), commonly referred to as gingival recession, denotes the reduction of gingival tissue along the gingival margin, resulting in the exposure of the tooth root. The occurrence of GR is attributed to a displacement of the gingival margin apical to the enamel-cementum junction, encompassing esthetic concerns, functional impairment, and various dental-related conditions [1]. Coronally advanced flap with connective tissue grafting is considered the gold standard in treating gingival recession, especially in cases of multiple adjacent teeth. However, this method may not be effective or sometimes contraindicated in the lower anterior region due to anatomical limitations and associated drawbacks [2]. In 1956, Grupe and Warren first introduced the sliding flap technique, and several studies improving this method have been published [3, 4].

## Case presentation

### Clinical examination and radiographic evaluation

**Figure 1:**
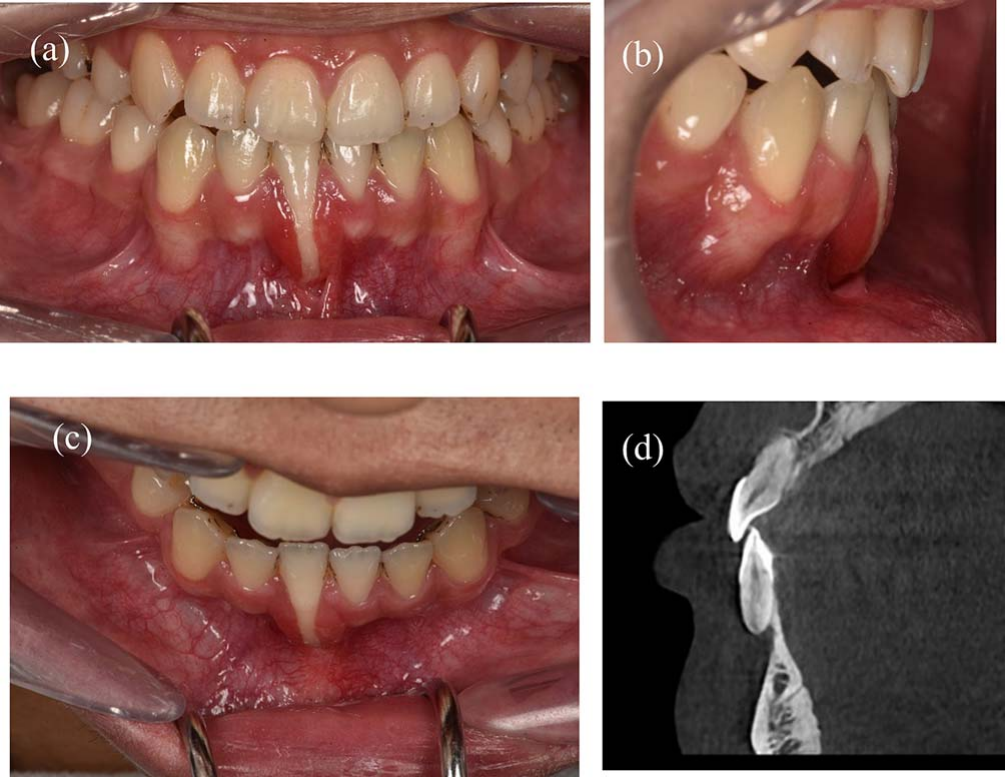
A 22-year-old man exhibits gingival recession in the lower left central incisor. (a) Intraoral image illustrating the labial view. (b) Intraoral image illustrating the lateral view. (c) Intraoral image illustrating the bird’s-eye dental view. (d) CBCT radiograph capturing the recession region.

A 22-year-old male patient exhibited an isolated buccal recession of Class I Cairo [5] on tooth 41, showing indications of significant inflammation in the marginal soft tissue. The patient had received a comprehensive orthodontic treatment 2 years ago. During the initial examination, there was a fixed lingual retainer extending from tooth 34 to tooth 44. The patient initially reported no recession of 41 after orthodontic treatment, but one year later, the recession appeared and became more serious after that. A fixed retainer can cause side effects on the teeth if it is not passive, leading to an undesirable force system and moment in the labial-lingual side (Fig. 1b) where the torque of tooth 41 showed a significant difference compared to other teeth. The periodontal assessment indicated the presence of a thin gingival phenotype, and a marginal tissue recession that exhibited a depth of ~9 mm, a width of 3 mm, and attached gingiva was absent. The measurement of marginal tissue recession was noted as the distance from the cemento- enamel junction to the tissue margin (Fig. 1a–c). Panoramic and cone beam computed tomography assessments were conducted. In the sagittal cross-sectional view of the cone beam computed tomography (CBCT) scan, the tooth exhibited malpositioning, extending beyond the dental alveolar bone (Fig. 1d).

### Treatment progress

After consulting with an orthodontic specialist, there was a consensus that it was a ‘wire syndrome’ [6, 7]; but not feasible to move the tooth back to its correct position because the bone was too thin. The aim of the treatment procedure was modify occlusion, undergo endodontic therapy, make alterations to the tooth structure, and execute soft tissue grafting to achieve complete coverage of the tooth root.

**Figure 2:**
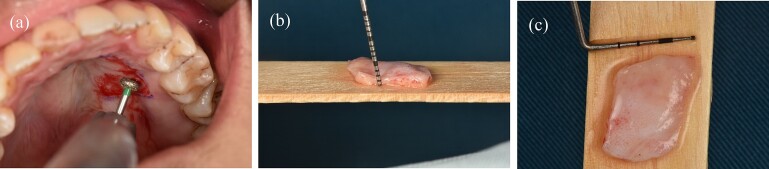
(a) The process of deepithelialization at the hard palate involves the utilization of a high-speed handpiece. (b) The thickness of a subepithelial connective tissue graft. (c) The dimensions of a subepithelial connective tissue graft.

**Figure 3:**
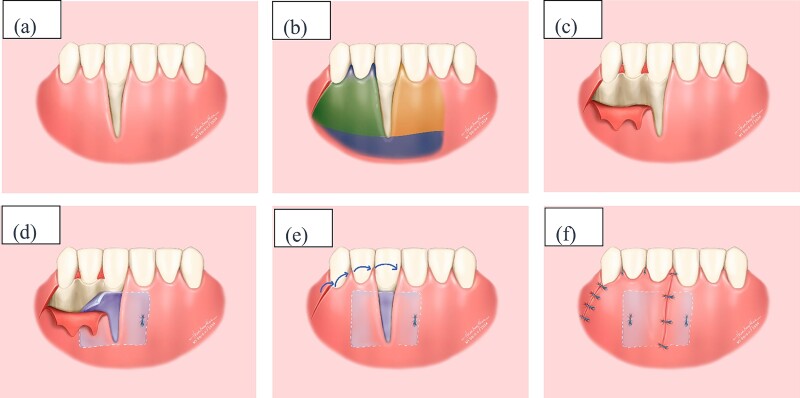
Illustrative depiction of the surgical procedure (a) Initial condition. (b) Flap design in the recipient area: yellow area represents tunnel incision, blue area represents split-thickness flap, and green area represents full-thickness flap. (c) Full-thickness flap. (d) Stabilize the connective tissue graft. (e) Rotate the flap and reposition the gingival margin. (f) Suture the flap.

**Figure 4:**
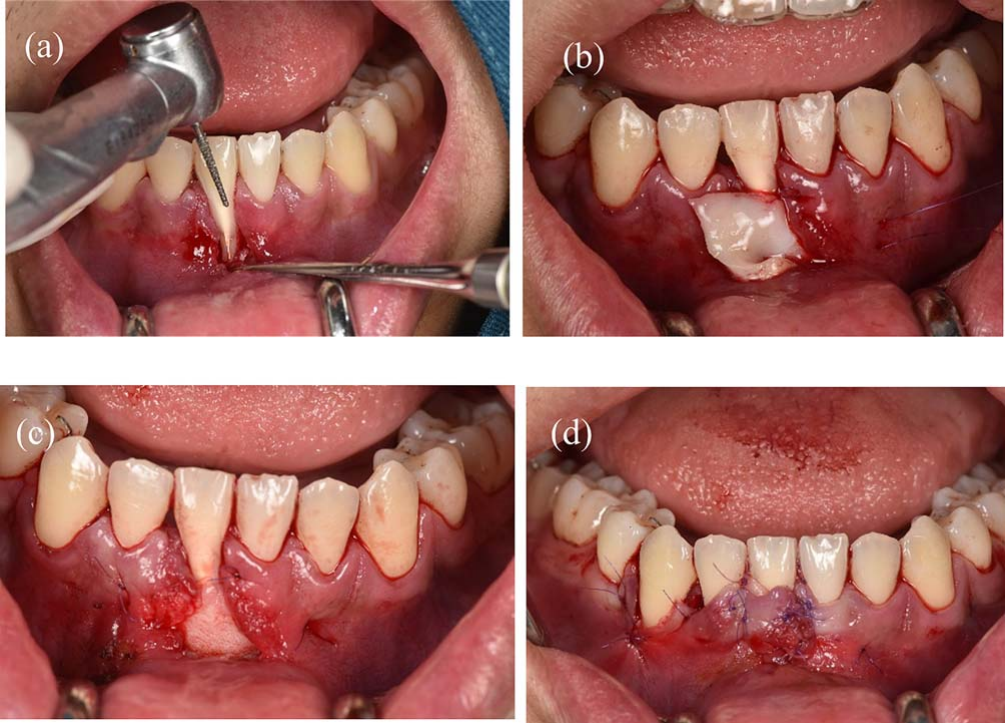
(a) Preparing the tooth surface using a high-speed handpiece. (b) Unilateral tunneling technique. (c) Stabilize the connective tissue graft. (d) Rotate the flap and reposition the gingival margin.

**Figure 5:**
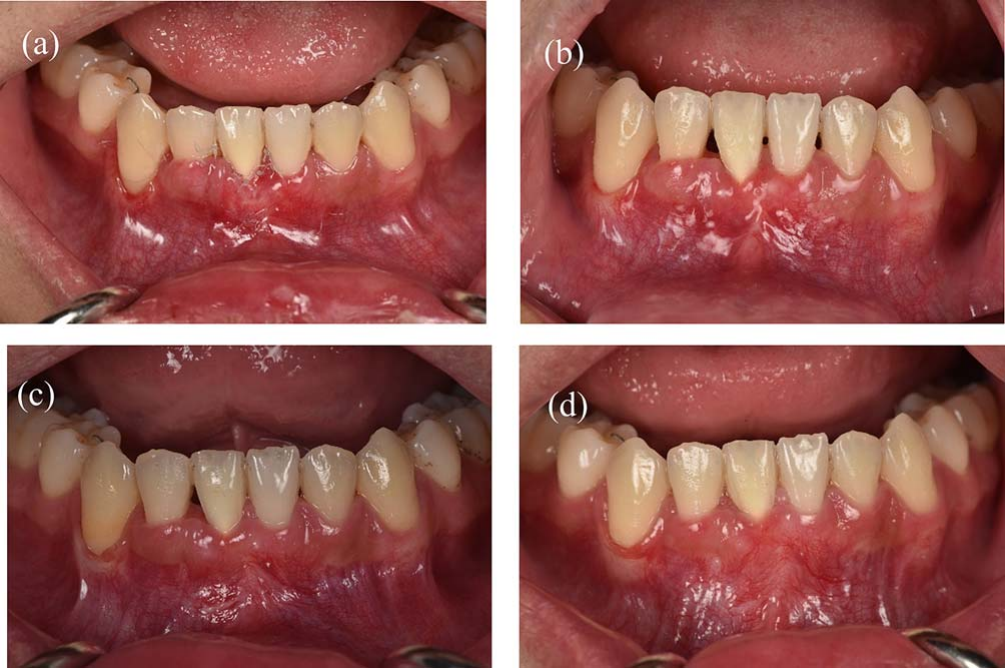
Clinical images of postoperative follow-up. (a) 3 weeks. (b) 2 months. (c) 6 months. (d) 12 months.

**Figure 6:**
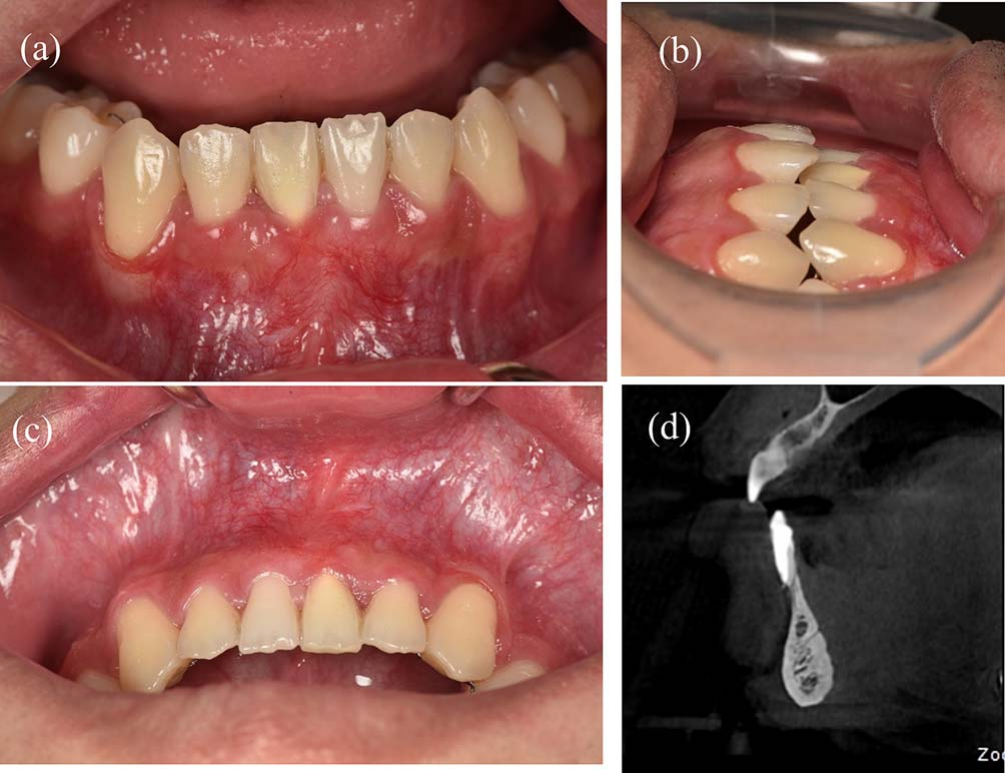
Results of treatment after one year of follow-up. (a) Intraoral image illustrating the labial view. (b) Intraoral image illustrating the lateral view. (c) Intraoral image illustrating the bird’s-eye dental view. (d) CBCT radiograph capturing the recession region.

Before surgery: scaled the lower incisors and endodontic treatment. After 2 weeks, the inflammation of the gingival had decreased and we can start the gingival graft treatment and the surgical procedure is depicted below (Fig. 3). Local anesthesia then followed by meticulous preparation of the tooth surface using a high-speed handpiece. To prepare the root surface, a 24% ethylenediaminetetraacetic acid (EDTA) gel was used. To obtain an epithelialized connective tissue graft (CTG), tissue was harvested from the hard palate in the area from first premolar to first molar. The graft was carefully shaped and sized based on the specific measurements of the gingival recession defect. A specialized 15C blade was utilized for this precise procedure. Subsequently, a thin layer of epithelial tissue, ~0.5–1 mm in thickness, was eliminated from the graft using a high-speed handpiece and 2 mm round diamond bur, except for the specified epithelial section as illustrated in (Fig. 2a). A piece of connective tissue in size 13 × 13 × 1.5 mm was harvested to adequately cover the entire tooth root (Fig. 2b and c). Afterward, Emdogain was applied to the complete root surface, starting from the most apical bone level to maximize the therapeutic potential of Emdogain in facilitating tissue regeneration and promoting periodontal healing. On the right side, where gingival recession was observed on the teeth, a flap with varying thickness was raised. This flap featured a split-thickness technique in the papillae and vestibular sulcus, while maintaining full thickness at the center. An incision was created to alleviate tension, specifically at a location distant to the right canine. Through the sulcular access on the left side of tooth 41, the graft was inserted into the tunnel, and the epithelial segment of the graft was positioned over the exposed root. Subsequently, the graft sites on both the left and right central incisors were secured using 6–0 monofilament sutures. Finally, the gingival flap was repositioned to cover the entire connective tissue area (Fig. 4). Subsequent assessments and recording of the healing process were performed at intervals of 3 weeks, 2 months, 6 months, and 1 year through reevaluation and imaging (Figs 5 and 6).

## Literature review and discussion

Gingival recession is most frequently observed in the mandibular incisors (43%) compared with other teeth [8]. Managing extensive labial gingival recessions in the anterior mandibular region poses a significant clinical challenge, primarily due to anatomical factors such as the elevated placement of the labial frenum, muscular fibers, restricted vestibular depth, thin tissues, and the absence of keratinized mucosa [9, 10]. There are various causes and influencing factors contributing to gingival recession, encompassing anatomical, pathological, oral hygiene, and iatrogenic factors [11]. It is important to note that orthodontic tooth movement should not be considered the primary cause of gingival retraction. The finer the cortical plate and marginal gingiva, the higher the probability of gingival retraction due to mechanical forces imposed by tooth brushing and/or the accumulation of dental plaque [12].

In 2018, Michele Agusto utilized the gingival pedicle with split-thickness tunnel technique for the treatment of single deep recessions on mandibular incisors treatment [4]. However, in the mandibular incisor region with a frequent occurrence of thin phenotype, the application of the split-thickness tunnel technique might present procedural challenges or feasibility issues during surgery. During orthodontic treatment, torque control [13] is important to avoid gingival recession, especially in open bite cases [14]. To achieve the goal of the treatment in open bite cases, we need to extrude the upper and lower incisors. Good biomechanics are necessary to control the treatment, and patient compliance is essential if elastics need to be worn. Sometimes, gingival graft after orthodontic treatment and a multidisciplinary approach may be necessary to achieve a successful result [15].

## Conclusion

In this case report, we discussed a technique for managing severe gingival recession after orthodontic treatment. The presented technique has consistently shown root coverage in the treatment of single deep narrow recession. Further investigation through ongoing research and comparative clinical studies will be essential to confirm these clinical observations. However, more research work and long-term follow-up will be needed to confirm the effectiveness.
